# Tubulin Inhibitors: A Chemoinformatic Analysis Using Cell-Based Data

**DOI:** 10.3390/molecules26092483

**Published:** 2021-04-24

**Authors:** Edgar López-López, Carlos M. Cerda-García-Rojas, José L. Medina-Franco

**Affiliations:** 1 Departamento de Química y Programa de Posgrado en Farmacología, Centro de Investigación y de Estudios Avanzados del Instituto Politécnico Nacional, Apartado 14-740, Mexico City 07000, Mexico; elopez.lopez@cinvestav.mx; 2 DIFACQUIM Research Group, Department of Pharmacy, School of Chemistry, Universidad Nacional Autónoma de México, Mexico City 04510, Mexico

**Keywords:** activity landscape, analog series, chemical space, cell-based assays, chemoinformatics, drug discovery, constellation plots, microtubules, scaffold, structure–property relationships

## Abstract

Inhibiting the tubulin-microtubules (Tub-Mts) system is a classic and rational approach for treating different types of cancers. A large amount of data on inhibitors in the clinic supports Tub-Mts as a validated target. However, most of the inhibitors reported thus far have been developed around common chemical scaffolds covering a narrow region of the chemical space with limited innovation. This manuscript aims to discuss the first activity landscape and scaffold content analysis of an assembled and curated cell-based database of 851 Tub-Mts inhibitors with reported activity against five cancer cell lines and the Tub-Mts system. The structure–bioactivity relationships of the Tub-Mts system inhibitors were further explored using constellations plots. This recently developed methodology enables the rapid but quantitative assessment of analog series enriched with active compounds. The constellations plots identified promising analog series with high average biological activity that could be the starting points of new and more potent Tub-Mts inhibitors.

## 1. Introduction

**Figure 1:**
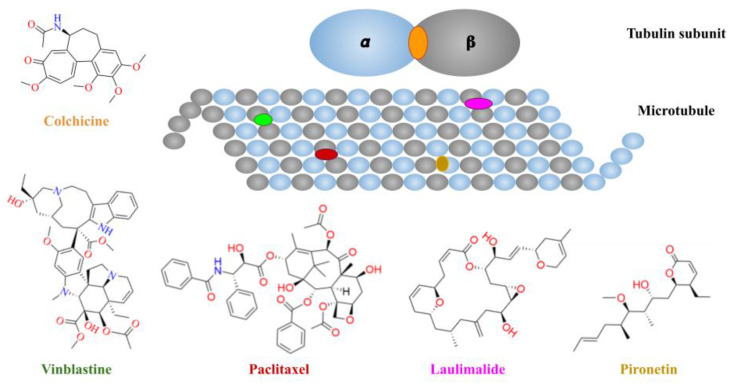
Schematic representation of the tubulin-binding sites on microtubules and chemical structures of representative inhibitors of the Tub-Mts system [2].

The α,β-tubulin heterodimer is the basic structural unit of microtubules. It is one of the most studied cancer therapy targets due to its significant role in cellular and tumor proliferation. It actively participates in forming the centrosome, an essential organelle, during the G_2_/M phase of the cell cycle [1]. The microtubule’s dynamic activity is guided by a polymerization and depolymerization process which can be modified by interaction with small molecules with different binding sites on the Tub-Mts system, e.g., colchicine, taxanes, pironetin, vinca alkaloids, and laulimalide derivatives, as shown in Figure 1. In this way, the modulation of polymerization/depolymerization of the microtubules allows for pharmacological regulation of the cell cycle, which is a crucial event in cancer [2]. According to the U.S. National Institute of Health (www.clinicaltrials.gov, accessed on 23 April 2021), there are several ongoing clinical studies in different phases related to tubulin inhibition: I (1604 studies), II (3771 studies), III (1410 studies), and IV (182 studies), that are analyzing colchicine derivatives (e.g., ombrabulin), taxanes (e.g., docetaxel), vinca alkaloids (e.g., ALB 109564) or laulimalide derivatives (e.g., epothilone D and eribulin). Although currently there are no pironetin analogs in clinical trials, pironetin is the first compound found to have the ability to covalently bind to microtubules, which gives it the capacity to inhibit the growth of cancer cells that are resistant to conventional treatments (derivatives of the vinca or paclitaxel) [3]. The small molecules are of synthetic, semi-synthetic, or natural origin. Figure 1 shows that the main binding sites are distributed along the microtubule. Additionally, the flexibility of microtubules’ quaternary structure has limited classical structure-based drug design approaches, such as rigid molecular docking. Several manuscripts that report predictive quantitative structure–activity relationships (QSAR) and machine learning models for compounds interacting with the Tub-Mts system have been published [4,5,6,7,8,9,10,11]. These studies have focused on the biological activity measured in biochemical assays. However, there are no reports on the quantitative analysis of the SAR of Tub-Mts system modulators tested in cell-based assays.

One consistent approach to characterize the SAR of compound datasets is through the systematic pairwise comparison of the structure with the activity. This approach, termed “activity landscape modeling” [12,13], is based upon the similarity principle, i.e., structurally similar compounds have similar activity. Activity landscape modeling can be generalized to “property landscape modeling”, where “property” includes a biological activity measured in vitro biochemical assays, cell-based, or any other type of activity with a measurable outcome. This approach identifies activity cliffs (AC), i.e., pairs of compounds with high structural similarity but large potency differences [14]. Depending on the work scope, an AC, which constitutes a significant exception to the similarity principle, can have beneficial or detrimental effects. For instance, an AC leads directly to essential structural information that influences the activity (property) [14]. Several quantitative and/or visual approaches have been published to characterize the activity (property) landscapes [15] of compounds with one or several endpoints over the past few years.

**Figure 2:**
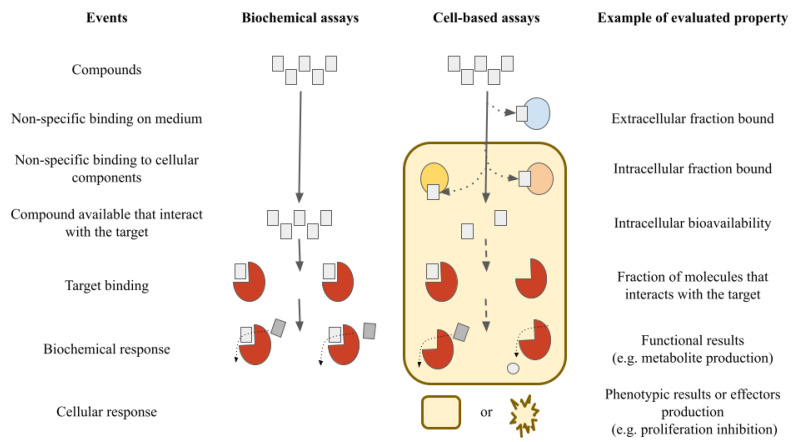
Schematic overview of the differences between biochemical and cell-based assays on the hit-to-lead process for drug discovery. Adapted from Mateus et al. [17].

Virtual screening methods initially deal with many compounds, until they are reduced to a manageable quantity [16]. Each type of evaluation has its challenges and complexity in regard to in vitro assays (e.g., biochemical or cell-based assays). It is not uncommon that compounds which are active in biochemical assays are inactive in cell-based assays. Each system (biochemical or cell-based assays) allows for analyzing the properties of different compounds, evaluating their pros and cons depending on the costs and how representative each system can be for the proposed study. This is shown schematically in Figure 2 [17]. 

A common and significant issue in the early phases of drug discovery, particularly in large screening campaigns, is the large number of false positives. This large number can be reduced by analyzing cell-based data instead of biochemical data. However, cell-based data do not provide direct information concerning the specific mechanism of action [17]. SAR studies are typically performed using activity obtained from biochemical assays, with quantitative measures of half-maximal inhibitory concentration (IC_50_), inhibitory constant (K_i_), or the percentage of inhibition [18]. The same happens in the particular case of activity (property) landscape studies [19]. Given that sizable cellular diversity exists and there are significant differences in the outcomes of biochemical and cell-based data analyses, the present study uses cell-based inhibition information to characterize the activity landscape of a herein generated dataset of Tub-Mts inhibitors reported in the literature.

The main goal of this work was to explore and describe the activity landscape and scaffold content [19] of a database built and curated herein, with 851 Tub-Mts inhibitors tested on cell-based assays and reported in the literature. To achieve the main goal, we analyzed the chemical space, structural chemical diversity, and scaffold content of the Tub-Mts inhibitors’ dataset.

## 2. Materials and Methods

### 2.1. Dataset

We assembled a dataset of 851 compounds tested as Tub-Mts inhibitors and with reported bioactivity in different cancer cell lines [20,21,22,23,24,25,26,27,28,29,30,31,32,33,34,35,36,37,38,39,40]. All compounds were retrieved from original and review articles and patents over a period of 15 years (2005–2020). The list of information sources is shown in Table S1 in the Supplementary Materials. The compounds were classified based on the bioactivity on different types of cancer cell lines: cervix (HeLa), colon (HCT-116), breast (MCF-7 and MDA-MB-231), lung (A-549), or prostate (PC3). The canonical SMILES [41] of the structures and pIC_50_ (−log IC_50_) values are listed in Table S1 in the Supplementary Materials. Duplicate molecules were removed using Molecular Operating Environment (MOE) software, version 2019 [42].

### 2.2. Chemical Space

Standard 2D chemical features were used to characterize the chemical space. The chemical space analysis focused on six physicochemical properties (PCP) of pharmaceutical relevance: octanol/water partition coefficient (cLog P), molecular weight (MW), topological polar surface area (TPSA), number of rotatable bonds (RB), number of hydrogen bond donors, and number of hydrogen bond acceptors (HBD/HBA). PCP-based clustering using t-distributed stochastic neighbor embedding (t-SNE) [43] was generated with DataWarrior, version 5.2.1 [44].

### 2.3. Activity Landscape Modeling

A structure–activity similarity (SAS) map is a two-dimensional graph suited for SAR analysis of compound datasets tested against a molecular target or a biological outcome. SAS maps are based on the concept of the activity landscape. They are suited for the rapid identification of AC, defined as compounds with a high structural similarity but unexpected large activity differences [45]. SAS maps also enable one to identify scaffold hops (SH), defined as compounds with low structural similarity due to differences in their scaffold but similar biological activity [45].

SAS maps were generated via systematic pairwise comparisons of the 851 compounds tested as Tub-Mts inhibitors. The structural similarity was calculated with the extended connectivity fingerprint 4 (ECFP4) (systematically records the neighborhood of each hydrogen atom at a distance of 4 bonds, for each atom within a molecule) [46] and the Tanimoto coefficient. This was represented on the *X*-axis to generate the map. The activity difference (pIC_50_ differences between each pair of compounds, e.g., if the pIC_50_ of compound “A” is 5 and the pIC_50_ of compound “B” is 7, the difference in activity of the pair of compounds A–B would be 2) was plotted on the *Y*-axis. The four major regions in the SAS map were defined using thresholds along the *X*- and *Y*-axis. There are several rational approaches to determine the thresholds [47]. In this work, the criteria to select the *X*-axis threshold was the “mean + two standard deviations” of the similarity values of compounds in the dataset (calculated with Tanimoto and the ECFP4 fingerprint). The threshold of the activity difference (*Y*-axis) was set to two logarithmic units [48]. 

The data points in the SAS map were further colored by the corresponding Structure–Activity Landscape Index (SALI) value [47]. This index, as implemented in Activity Landscape Plotter [47], quantifies AC using the equation proposed by Guha and Van Drie:

*SALII*,*J* = ((|*Ai* − *Aj*|)/(1 − *sim*(*i*,*j*)))

where *Ai* and *Aj* are the activities of the *i*th and the *j*th molecules, and *sim* (*i*,*j*) is the similarity coefficient between the two molecules (in this work, computed with the ECFP4 fingerprint and the Tanimoto coefficient). Quantitative analysis of the SAS maps was done with Activity Landscape Plotter, a web server freely available at http://132.248.103.152:3838/ActLSmaps/ (accessed on 23 April 2021) [48].

### 2.4. Scaffold Content Analysis

To study the molecular scaffolds, we used the methodology implemented by Bemis and Murcko [49]. Briefly, the method involves a graph analysis for each compound where a “scaffold” is defined as the union of ring systems and linkers in a molecule, and the side chains are removed (any non-ring, non-linker atoms). This was done with the RDKit Fragments node implemented in KNIME, version 3.7.2 [50]. The chemical structures of the scaffolds are available in Table S2 of the Supplementary Materials.

### 2.5. Constellation Plot

A constellation plot is a graphical representation of chemical space based on networks and coordinates. Each node represents a group of chemical analog series. In other words, a constellation plot reduces the number of points depicted in chemical space representations, while increasing the quality and volume of data legibly represented in a 2D chemical space plot. They aim to group analogs that share a common core, which can be compared with other cores and their corresponding associated molecules [51]. A constellation plot was generated using free Python code published elsewhere [52]. RDKit was used for computing Morgan fingerprints and to handle the chemical structures (http://www.rdkit.org, accessed on 23 April 2021); Scikit-learn was used for computing the t-SNE (scaffold-based clustering). The output file was adapted to be viewed interactively using the DataWarrior software (see the file “out_FinalData.dwar” in the Supplementary Materials). This is the first interactive constellation plot reported so far.

## 3. Results and Discussion

As mentioned in the Introduction Section, most of the Tub-Mts system’s reported inhibitors are derivatives of four main principal structures, as shown in Figure 1. Thus, to further develop the inhibitors of the Tub-Mts system, it is common to first perform cell-based screening, followed by a screening using a biochemical assay (i.e., tubulin polymerization) of the most active compounds. The new series of compounds lack information about the mechanism of action and the specific binding site in tubulin. Therefore, we employed chemoinformatic approaches to explore the SAR of 851 compounds with reported cell-based inhibitory activity.

Although there are advantages to using data based on cell line inhibition, there are also disadvantages. For example, the bioactivity results reported in the literature depend on the type of test and protocol used. Hence, it is not possible to compare each compound directly against another, and approximations have to be made to explore the SAR. In this scenario, this work aims to fill part of the information gaps left by conventional SAR and QSAR studies.

### 3.1. Chemoinformatics Approaches

#### 3.1.1. Chemical Space

**Figure 3:**
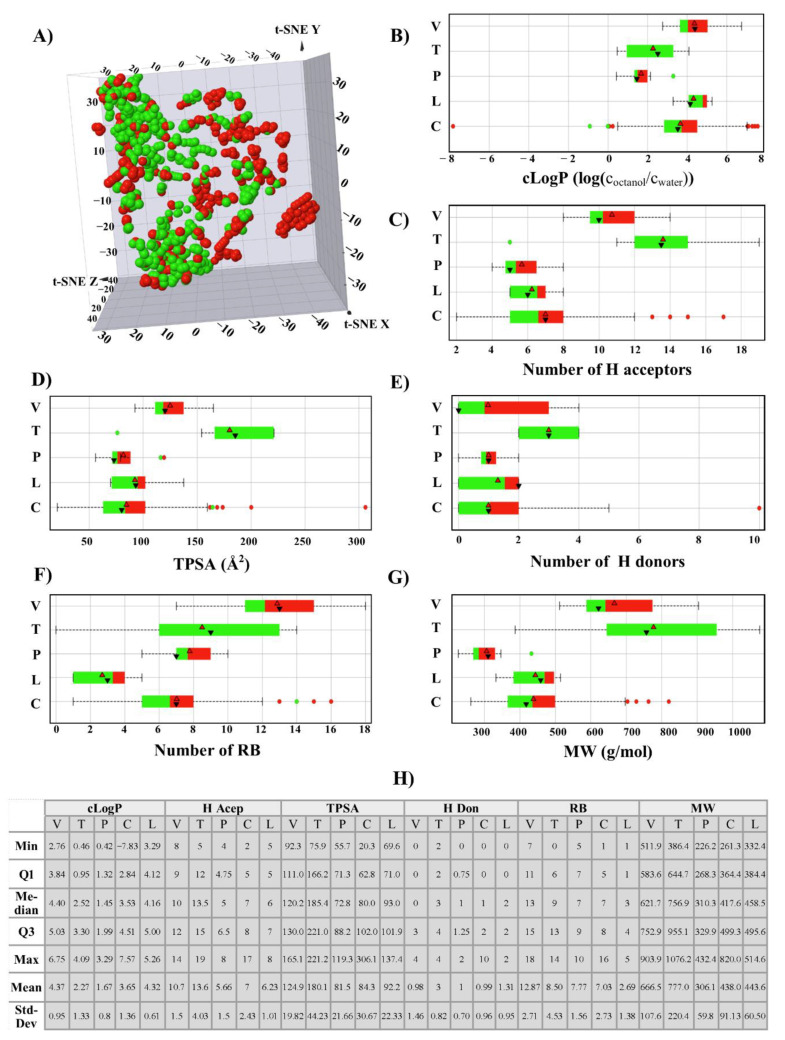
Overview of physicochemical properties of 851 compounds tested in cell-based assays. Compounds are colored by ranges of biological activity values: Green as active (<1 µM) and red as less-active (>1 µM). The compounds were grouped by binding site: vinca site (V), paclitaxel site (T), pironetin (P), laulimalide (L), and colchicine site (C). (**A**) Visual representation of the chemical space using t-SNE; (**B**–**G**) Box plots of the drug-like properties commonly used in the drug design and development process. (**B**) cLog P; (**C**) number of H bond acceptors; (**D**) TPSA (topological surface area); (**E**) number of H bond donors; (**F**) RB (rotatable bonds); (**G**) MW (molecular weight). The boxes enclose data points with values in the first and third quartile. The red and black triangles denote the mean and median distributions, respectively. The data points outside of the boxes indicate outliers; (**H**) Summary statistics of the dataset.

Figure 3A shows a visual representation of the chemical space of the 851 compounds using t-SNE coordinates based on the six PCP of pharmaceutical interest described in the Materials and Methods Section. The box plot analysis of the common drug-like properties for drug discovery is shown in Figure 3B–G. The figure shows the data summarized using the information presented by the box plots. Such as, in panel B (cLog P), the active compounds (green area) have values of around 2.5 to 4.0, and the inactive compounds (red area) have higher values. This information can be used to generate new knowledge about this kind of inhibitor, e.g., generally, compounds with cLog P values higher than four are inactive. It is important to note that the average PCP values of compounds are different, depending on the tubulin’s binding site. For example, the cLog P values of vinca-like inhibitors (e.g., vinblastine derivatives) are higher than those of colchicine-like inhibitors; in other words, vinca derivatives are more lipophilic than colchicine derivatives. This can be deduced from the higher number of aromatic and non-aromatic rings of vinblastine than colchicine (Figure 1).

#### 3.1.2. Activity Landscape Modeling

**Figure 4:**
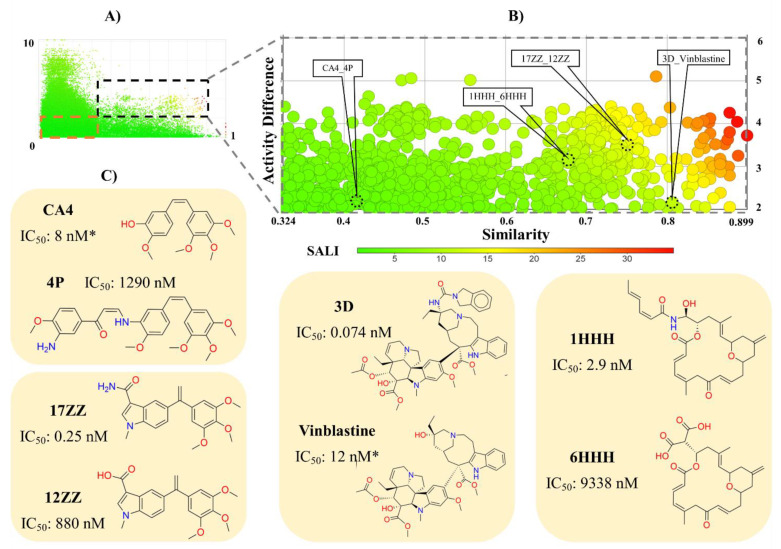
Structure-activity similarity (SAS) map of compounds with activity against Tub-Mts system using cell-based inhibition data. (**A**) General activity landscape of compounds. The activity cliffs and scaffold hops zone was defined by a black and red dotted line, respectively; (**B**) activity cliff zone. Data points are colored by SALI value using a continuous scale from low (green) to high (red); (**C**) representative activity cliffs. Activity marked as (*) is a representative value of the activity reported in different cell lines. The similarity was calculated with the Tanimoto coefficient and the ECFP4 fingerprint.

Following the activity landscape concept described in the Introduction and Methods Sections, we analyzed the SAR of the 851 compounds tested in cell-based assays. Figure 4A,B shows the SAS map and amplification on the AC zone, respectively. Each point represents a pair of compounds that are colored by SALI values, using a color scale from green (low SALI value, i.e., non-AC) to red (high SALI value, i.e., AC). The information helps identify small structural changes in molecules that decrease or increase their bioactivity. Interestingly, 97% of the data points correspond to a series of compounds with low structural similarity: 67% are SH (compounds with low structural similarity and low activity differences or high activity similarity). This result is indicative of the high structural diversity of the compounds in this dataset. Figure 4B,C depicts the chemical structures of representative AC. 

**Figure 5:**
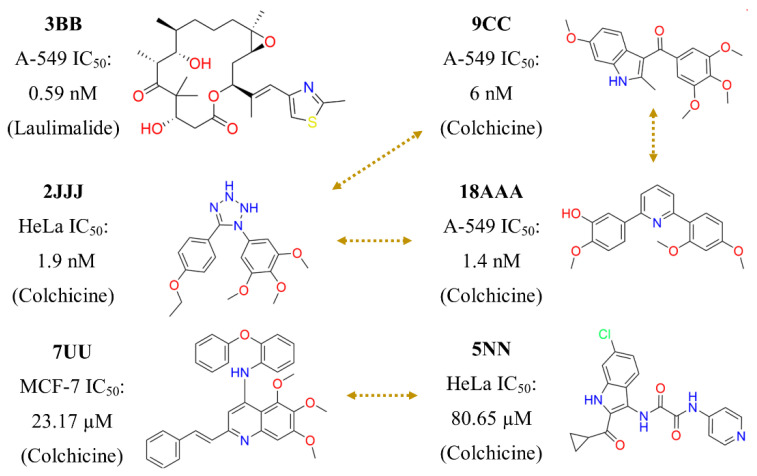
Representative diversity of compounds and scaffolds content on the database. These compounds were selected from the scaffold hops identified in the activity landscape analysis (Figure 4). The dotted arrows connect compounds with similar activity (which interact with the same binding site) but different chemical scaffolds. The binding site of each compound is shown in parentheses.

We emphasize that the bioactivity data were derived from cell-based assays. Therefore, it involves the affinity of these compounds with the main target (Tub-Mts system) and also with the ability to interact favorably with a biological (cellular) system, e.g., consider the membrane barriers and non-specific bindings (see Introduction Section). In addition to Figure 1,Figure 4 and Figure 5 illustrates other representative compounds and scaffolds with bioactivity reported against the Tub-Mts system. Except for compound **3BB** (designed to interact with the laulimalide binding site), all other compounds shown were designed to interact with the colchicine binding site. However, as emphasized above, the precise binding site remains to be elucidated using biophysical assays.

#### 3.1.3. Scaffold Content Analysis

**Figure 6:**
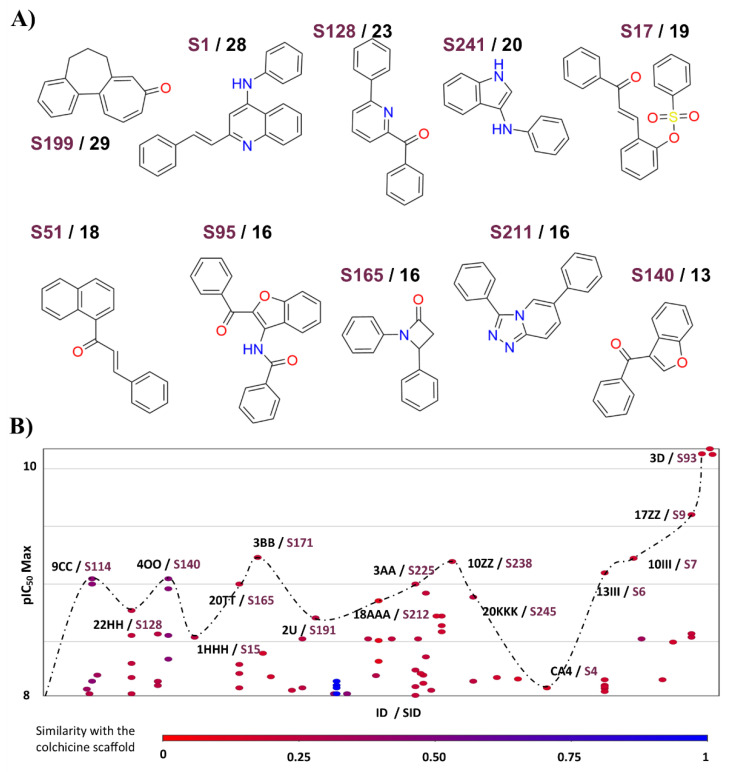
Scaffold content analysis of compounds with activity against Tub-Mts system using cell-based inhibition data. (**A**) Frequency of most common scaffolds. The scaffolds were generated using Bemis and Murcko’s definition; (**B**) scaffolds of the most active compounds and activity differences. The pIC_50_ values plotted were the most active values reported independently of the cell line (pIC_50_ Max). Each compound identifier (**ID**) and each scaffold identifier (**SID**) are shown for each structure. Each scaffold is colored by the low (red) or high (blue) similarity value to the colchicine’s Bemis and Murcko scaffold.

Figure 6A shows an overview of the ten most common scaffolds of compounds tested in cell-based assays against the Tub-Mts system. In contrast, Figure 6B shows a landscape with the most bioactive compounds’ IDs (and their respective scaffold identifiers). Figure 6B plots the pIC_50_ Max values (maximum IC_50_ value reported in any cell line) in relationship with a list of compounds (and its respective scaffolds) represented by each point. The dotted line represents the “cliff path” of activity from one compound (and its respective scaffolds) to another. For example, compound **3D** is more bioactive (pIC_50_ Max = 10.1) than **17ZZ** (pIC_50_ Max = 9.6), and the latter, in turn, is more bioactive than **10III** (pIC_50_ Max = 9.2). In Figure 6B, the compounds (represented by points) are colored by a color scale, from red (lower similarity value to the colchicine scaffold) to blue (higher similarity to the colchicine scaffold).

Interestingly, only **S128**, **S140**, and **S165** (common scaffolds) are contained in five, four, and four cases, respectively, of the most active compounds. Representative examples of the most active compounds with common scaffolds are **22HH, 4OO,** and **20TT** (see Figure S1 in the Supplementary Materials which illustrates other active compounds that were not found in the AC or scaffold hops sections). In other words, they are scaffolds (Bemis–Murcko) that are not necessarily contained in the most active compounds. The complete list of scaffolds is in Table S2 in the Supplementary Materials.

#### 3.1.4. Constellation Plot

**Figure 7:**
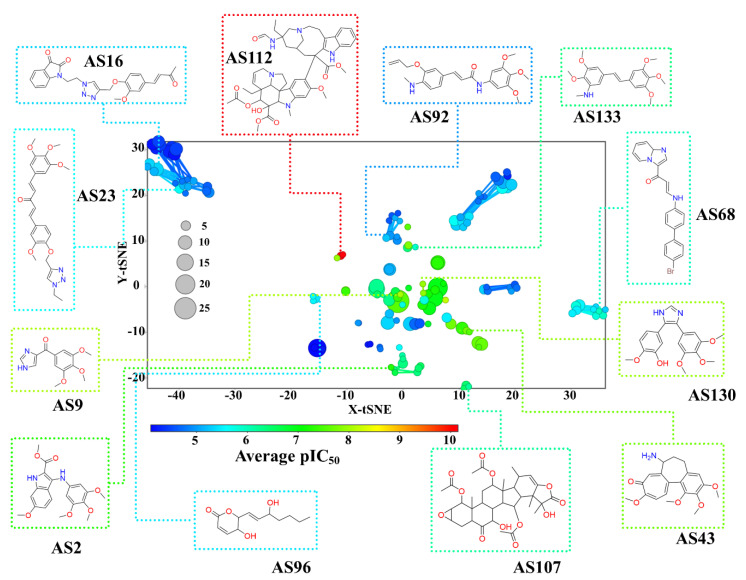
Constellation plot of compounds with activity against Tub-Mts system using cell-based inhibition data. The plot shows 147 data points, each one representing an analog series. Selected analog series are identified with a number. The data point’s size indicates the relative number of compounds in each analog series, and the color is the average activity of the compound in the series.

Constellation plots were developed as a graphical representation of SAR to integrate coordinate-based chemical space representation with analog series. These plots summarize the scaffolds content of a dataset and show the scaffold diversity and their mutual structural relationships [51]. Since the biological activity data can be mapped into a constellation plot, these two-dimensional representations of the chemical space enable the identification of whole regions in chemical space rich in SAR annotations, either active (“bright” SAR in analogy with chemical space) or inactive (“dark regions”), where few or no active molecules have been found. Figure 7 shows the constellation plot of Tub-Mts inhibitors (an interactive version of the plot that can be visualized with DataWarrior and is available in the Supplementary Materials).

From the initial compound database, the chemical structures of 851 inhibitors have been summarized into 142 analog series (illustrated in Figure 7), which further summarize the 258 Bemis–Murcko scaffolds in Table S2 (Supplementary Materials). Of note, compounds with different Bemis–Murcko scaffolds share a structural fraction (that is not a complete scaffold). This explains why molecules with different scaffolds are contained in the same analog series. An analog series considers the synthetic route to generate the compounds which are based on RECAP (retrosynthetic rules) [53]. In contrast, Bemis–Murcko scaffolds do not consider synthetic rules; these remove each compound’s side chains. Additionally, the constellation plots order the analog series using similarity-based coordinates, i.e., analog series with similar chemical structures are closely ordered because they share similar X and Y coordinates in the 2D plots. In contrast, analog series with more different chemical structures remain far apart. As mentioned in the Materials and Methods Section, the similarity and coordinate data are not comparable between Figure 4A,B (calculated similarity between pairs of compounds) and Figure 7 (calculated similarity between analog series).

The analog series ID, SMILES, coordinates (t-SNE coordinates), average activity (average of pIC_50_), compounds contained per analog series, and standard deviations are included in the file “out_FinalData.dwar” in the Supplementary Materials.

Figure 7 illustrates representative “dark” and “bright” inhibitors of chemical space. Each point in the graph corresponds to a complete analog series and the data points are colored by the average pIC_50_ of all compounds in that particular analog series. The average activity is colored using a scale from blue (less active) to red (more active). The size of each point represents the relative number of molecules contained in the analog series. Additionally, linking lines represent shared molecules between two analog series.

The constellation plot in Figure 7 shows the clear identification of “dark regions” in the SAR in the chemical space of Tub-Mts inhibitors, e.g., the analog series in light blue with low average pIC_50_ values: **AS16**, **AS23**, **AS68, AS92** and **AS96**. The plot also aids the identification of promising analog series or “emerging bright stars” in the chemical space, e.g., the analog series in green-to-red color with high average pIC_50_ values, **AS2**, **AS9**, **AS43**, **AS112**, **AS130,** and **AS133**. Although these analog series have been explored on a limited basis (between two and three compounds, as depicted by the smaller size of the data point in Figure 7) they have a high average activity. Thus, these analog series could be the future of new and potent inhibitors. However, and despite their high average activity, some series (e.g., **AS112**) could still have limitations such as their difficult synthetic accessibility or poor pharmacokinetic profile.

Figure S2 in the Supplementary Materials shows a summary of the major analog series (constellations in chemical space) related to the chemical structures of four principal inhibitors of the Tub-Mts system (illustrated in Figure 1). The analog series (constellations) with labels **AS16**, **AS23**, **AS68,** and **AS112** in Figure 7 are analog series of compounds that interact with the vinca binding site. The analog series **AS2**, **AS9**, **AS43, AS130**, and **AS133** in Figure 7 interact with the colchicine binding site. Other representative examples are the analog series **AS96** and **AS107** (Figure 7), which include compounds that interact with the pironetin and paclitaxel binding sites, respectively. Of note, there were no analog series for inhibitors that interact with the laulimalide binding site since none of them complied with the retrosynthesis rules (RECAP) considered in this work, so they are not visualized in the constellation plot.

## 4. Conclusions

The present work explored and described the first activity landscape and scaffold content analysis of a newly assembled and curated cell-based database of 851 Tub-Mts inhibitors with reported activity against five cancer cell lines and the Tub-Mts system. The study revealed that the current Tub-Mts inhibitors are a series of compounds with limited molecular and scaffold diversity. It was also concluded that there are differences in the physicochemical profile that depend on the inhibitors’ binding site (e.g., against colchicine, vinca, pironetin, paclitaxel, or laulimalide binding site). Cell-based data implicitly contain information that is not possible to analyze via biochemical assays. We propose using this information to generate SAR and QSAR predictive methods to reduce the error rate in biological evaluations of novel inhibitors of the Tub-Mts systems. Additionally, Tub-Mts system’s inhibitors were explored using constellation plots; this novel visualization of the SAR of chemical datasets led to the identification of promising analog series with high average pIC_50_ values (e.g., **AS2**, **AS9**, **AS43**, **AS112**, **AS130**, and **AS133**); these analog series could be the starting point of new and potent Tub-Mts inhibitors.

## Data Availability

Not applicable.

## Supplementary Materials

The following are available online, Table S1: Compound dataset, Table S2: List of Bemis–Murcko scaffolds, Figure S1: Active compounds that were not found in the AC or scaffold hops sections, Figure S2: Summary of major analog series related to the chemical structures of four principal inhibitors by the Tub-Mts system, out_FinalData.dwar: Interactive output to be viewed using the DataWarrior software.

## References

[1] Arnst K.E., Banerjee S., Chen H., Deng S., Hwang D., Li W., Miller D.D. (2019). Current Advances of Tubulin Inhibitors as Dual Acting Small Molecules for Cancer Therapy. Med. Res. Rev..

[2] Lu Y., Chen J., Xiao M., Li W., Miller D.D. (2012). An Overview of Tubulin Inhibitors That Interact with the Colchicine Binding Site. Pharm. Res..

[3] Coulup S.K., Georg G.I. (2019). Revisiting Microtubule Targeting Agents: α-Tubulin and the Pironetin Binding Site as Unexplored Targets for Cancer Therapeutics. Bioorg. Med. Chem. Lett..

[4] Chen H., Deng S., Wang Y., Albadari N., Kumar G., Ma D., Li W., White S.W., Miller D.D., Li W. (2020). Structure–Activity Relationship Study of Novel 6-Aryl-2-Benzoyl-Pyridines as Tubulin Polymerization Inhibitors with Potent Antiproliferative Properties. J. Med. Chem..

[5] Xu Q., Bao K., Sun M., Xu J., Wang Y., Tian H., Zuo D., Guan Q., Wu Y., Zhang W. (2017). Design, Synthesis and Structure-Activity Relationship of 3,6-Diaryl-7H-[1,2,4]Triazolo[3,4-b][1,3,4]Thiadiazines as Novel Tubulin Inhibitors. Sci. Rep..

[6] Ducki S., Mackenzie G., Lawrence N.J., Snyder J.P. (2005). Quantitative Structure−Activity Relationship (5D-QSAR) Study of Combretastatin-like Analogues as Inhibitors of Tubulin Assembly. J. Med. Chem..

[7] Fu D.-J., Fu L., Liu Y.-C., Wang J.-W., Wang Y.-Q., Han B.-K., Li X.-R., Zhang C., Li F., Song J. (2017). Structure-Activity Relationship Studies of β-Lactam-Azide Analogues as Orally Active Antitumor Agents Targeting the Tubulin Colchicine Site. Sci. Rep..

[8] Aziz J., Brachet E., Hamze A., Peyrat J.-F., Bernadat G., Morvan E., Bignon J., Wdzieczak-Bakala J., Desravines D., Dubois J. (2013). Synthesis, Biological Evaluation, and Structure–Activity Relationships of Tri- and Tetrasubstituted Olefins Related to Isocombretastatin A-4 as New Tubulin Inhibitors. Org. Biomol. Chem..

[9] Romagnoli R., Baraldi P.G., Carrion M.D., Cara C.L., Cruz-Lopez O., Tolomeo M., Grimaudo S., Cristina A.D., Pipitone M.R., Balzarini J. (2009). Design, Synthesis and Structure–Activity Relationship of 2-(3′,4′,5′-Trimethoxybenzoyl)-Benzo[b]Furan Derivatives as a Novel Class of Inhibitors of Tubulin Polymerization. Bioorg. Med. Chem..

[10] Mirzaei S., Hadizadeh F., Eisvand F., Mosaffa F., Ghodsi R. (2020). Synthesis, Structure-Activity Relationship and Molecular Docking Studies of Novel Quinoline-Chalcone Hybrids as Potential Anticancer Agents and Tubulin Inhibitors. J. Mol. Struct..

[11] Guo Q., Zhang H., Deng Y., Zhai S., Jiang Z., Zhu D., Wang L. (2020). Ligand- and Structural-Based Discovery of Potential Small Molecules That Target the Colchicine Site of Tubulin for Cancer Treatment. Eur. J. Med. Chem..

[12] Bajorath J., Peltason L., Wawer M., Guha R., Lajiness M.S., Van Drie J.H. (2009). Navigating Structure–Activity Landscapes. Drug Discov. Today.

[13] Medina-Franco J.L., Navarrete-Vázquez G., Méndez-Lucio O. (2015). Activity and Property Landscape Modeling Is at the Interface of Chemoinformatics and Medicinal Chemistry. Future Med. Chem..

[14] Maggiora G.M. (2006). On Outliers and Activity CliffsWhy QSAR Often Disappoints. J. Chem. Inf. Model..

[15] Maggiora G., Medina-Franco J.L., Iqbal J., Vogt M., Bajorath J. (2020). From Qualitative to Quantitative Analysis of Activity and Property Landscapes. J. Chem. Inf. Model..

[16] Sink R., Gobec S., Pecar S., Zega A. (2010). False Positives in the Early Stages of Drug Discovery. Curr. Med. Chem..

[17] Mateus A., Gordon L.J., Wayne G.J., Almqvist H., Axelsson H., Seashore-Ludlow B., Treyer A., Matsson P., Lundbäck T., West A. (2017). Prediction of Intracellular Exposure Bridges the Gap between Target- and Cell-Based Drug Discovery. Proc. Natl. Acad. Sci. USA.

[18] Mendez D., Gaulton A., Bento A.P., Chambers J., De Veij M., Félix E., Magariños M.P., Mosquera J.F., Mutowo P., Nowotka M. (2019). ChEMBL: Towards Direct Deposition of Bioassay Data. Nucleic Acids Res..

[19] López-López E., Rabal O., Oyarzabal J., Medina-Franco J.L. (2020). Towards the Understanding of the Activity of G9a Inhibitors: An Activity Landscape and Molecular Modeling Approach. J. Comput. Aided Mol. Des..

[20] Shen Y.-N., Lin L., Qiu H.-Y., Zou W.-Y., Qian Y., Zhu H.-L. (2015). The Design, Synthesis, in Vitro Biological Evaluation and Molecular Modeling of Novel Benzenesulfonate Derivatives Bearing Chalcone Moieties as Potent Anti-Microtubulin Polymerization Agents. RSC Adv..

[21] Ghawanmeh A.A., Al-Bajalan H.M., Mackeen M.M., Alali F.Q., Chong K.F. (2020). Recent Developments on (−)-Colchicine Derivatives: Synthesis and Structure-Activity Relationship. Eur. J. Med. Chem..

[22] Li W., Xu F., Shuai W., Sun H., Yao H., Ma C., Xu S., Yao H., Zhu Z., Yang D.-H. (2019). Discovery of Novel Quinoline–Chalcone Derivatives as Potent Antitumor Agents with Microtubule Polymerization Inhibitory Activity. J. Med. Chem..

[23] Kamal A., Kumar G.B., Polepalli S., Shaik A.B., Reddy V.S., Reddy M.K., Reddy C.R., Mahesh R., Kapure J.S., Jain N. (2014). Design and Synthesis of Aminostilbene-Arylpropenones as Tubulin Polymerization Inhibitors. ChemMedChem.

[24] Miao T.-T., Tao X.-B., Li D.-D., Chen H., Jin X.-Y., Geng Y., Wang S.-F., Gu W. (2018). Synthesis and Biological Evaluation of 2-Aryl-Benzimidazole Derivatives of Dehydroabietic Acid as Novel Tubulin Polymerization Inhibitors. RSC Adv..

[25] Wang G., Liu W., Gong Z., Huang Y., Li Y., Peng Z. (2020). Synthesis, Biological Evaluation, and Molecular Modelling of New Naphthalene-Chalcone Derivatives as Potential Anticancer Agents on MCF-7 Breast Cancer Cells by Targeting Tubulin Colchicine Binding Site. J. Enzym. Inhib. Med. Chem..

[26] Sankara Rao N., Lakshma Nayak V., Subba Rao A.V., Ali Hussaini S.M., Sunkari S., Alarifi A., Kamal A. (2019). Arylcinnamido-Propionone Conjugates as Tubulin Polymerization Inhibitors and Apoptotic Inducers. Arab. J. Chem..

[27] Wang C., Li Y., Liu T., Wang Z., Zhang Y., Bao K., Wu Y., Guan Q., Zuo D., Zhang W. (2020). Design, Synthesis and Evaluation of Antiproliferative and Antitubulin Activities of 5-Methyl-4-Aryl-3-(4-Arylpiperazine-1-Carbonyl)-4H-1,2,4-Triazoles. Bioorg. Chem..

[28] Yang F., Jian X.-E., Diao P.-C., Huo X.-S., You W.-W., Zhao P.-L. (2020). Synthesis, and Biological Evaluation of 3,6-Diaryl-[1,2,4]Triazolo[4,3-a]Pyridine Analogues as New Potent Tubulin Polymerization Inhibitors. Eur. J. Med. Chem..

[29] Li Q., Jian X.-E., Chen Z.-R., Chen L., Huo X.-S., Li Z.-H., You W.-W., Rao J.-J., Zhao P.-L. (2020). Synthesis and Biological Evaluation of Benzofuran-Based 3,4,5-Trimethoxybenzamide Derivatives as Novel Tubulin Polymerization Inhibitors. Bioorg. Chem..

[30] Huang L., Liu M., Man S., Ma D., Feng D., Sun Z., Guan Q., Zuo D., Wu Y., Zhang W. (2020). Design, Synthesis and Bio-Evaluation of Novel 2-Aryl-4-(3,4,5-Trimethoxy-Benzoyl)-5-Substituted-1,2,3-Triazoles as the Tubulin Polymerization Inhibitors. Eur. J. Med. Chem..

[31] Diao P.-C., Jian X.-E., Chen P., Huang C., Yin J., Huang J.C., Li J.-S., Zhao P.-L. (2020). Design, Synthesis and Biological Evaluation of Novel Indole-Based Oxalamide and Aminoacetamide Derivatives as Tubulin Polymerization Inhibitors. Bioorg. Med. Chem. Lett..

[32] Oliva P., Romagnoli R., Manfredini S., Brancale A., Ferla S., Hamel E., Ronca R., Maccarinelli F., Giacomini A., Rruga F. (2020). Design, Synthesis, in Vitro and in Vivo Biological Evaluation of 2-Amino-3-Aroylbenzo[b]Furan Derivatives as Highly Potent Tubulin Polymerization Inhibitors. Eur. J. Med. Chem..

[33] Shao Y.-Y., Yin Y., Lian B.-P., Leng J.-F., Xia Y.-Z., Kong L.-Y. (2020). Synthesis and Biological Evaluation of Novel Shikonin-Benzo[b]Furan Derivatives as Tubulin Polymerization Inhibitors Targeting the Colchicine Binding Site. Eur. J. Med. Chem..

[34] Romagnoli R., Prencipe F., Oliva P., Kimatrai Salvador M., Brancale A., Ferla S., Hamel E., Viola G., Bortolozzi R., Persoons L. (2020). Design, Synthesis and Biological Evaluation of 2-Alkoxycarbonyl-3-Anilinoindoles as a New Class of Potent Inhibitors of Tubulin Polymerization. Bioorg. Chem..

[35] Tang H., Cheng J., Liang Y., Wang Y. (2020). Discovery of a Chiral Fluorinated Azetidin-2-One as a Tubulin Polymerisation Inhibitor with Potent Antitumour Efficacy. Eur. J. Med. Chem..

[36] Mirzaei S., Eisvand F., Hadizadeh F., Mosaffa F., Ghasemi A., Ghodsi R. (2020). Design, Synthesis and Biological Evaluation of Novel 5,6,7-Trimethoxy-N-Aryl-2-Styrylquinolin-4-Amines as Potential Anticancer Agents and Tubulin Polymerization Inhibitors. Bioorg. Chem..

[37] Li G., Wang Y., Li L., Ren Y., Deng X., Liu J., Wang W., Luo M., Liu S., Chen J. (2020). Design, Synthesis, and Bioevaluation of Pyrazolo[1,5-a]Pyrimidine Derivatives as Tubulin Polymerization Inhibitors Targeting the Colchicine Binding Site with Potent Anticancer Activities. Eur. J. Med. Chem..

[38] Poornima B., Siva B., Venkanna A., Shankaraiah G., Jain N., Yadav D.K., Misra S., Babu K.S. (2017). Novel Gomisin B Analogues as Potential Cytotoxic Agents: Design, Synthesis, Biological Evaluation and Docking Studies. Eur. J. Med. Chem..

[39] Martino E., Casamassima G., Castiglione S., Cellupica E., Pantalone S., Papagni F., Rui M., Siciliano A.M., Collina S. (2018). Vinca Alkaloids and Analogues as Anti-Cancer Agents: Looking Back, Peering Ahead. Bioorg. Med. Chem. Lett..

[40] Maklad R.M., AbdelHafez E.-S.M.N., Abdelhamid D., Aly O.M. (2020). Tubulin Inhibitors: Discovery of a New Scaffold Targeting Extra-Binding Residues within the Colchicine Site through Anchoring Substituents Properly Adapted to Their Pocket by a Semi-Flexible Linker. Bioorg. Chem..

[41] Weininger D. (1988). SMILES, a Chemical Language and Information System. 1. Introduction to Methodology and Encoding Rules. J. Chem. Inf. Model..

[42] Chemical Computing Group Inc. (2020). Molecular Operating Environment (MOE).

[43] Van der Maaten L., Hinton G. (2008). Visualizing Data Using T-SNE. J. Mach. Learn. Res..

[44] López-López E., Naveja J.J., Medina-Franco J.L. (2019). DataWarrior: An Evaluation of the Open-Source Drug Discovery Tool. Expert Opin. Drug Dis..

[45] Medina-Franco J.L. (2012). Scanning Structure–Activity Relationships with Structure–Activity Similarity and Related Maps: From Consensus Activity Cliffs to Selectivity Switches. J. Chem. Inf. Model..

[46] Rogers D., Hahn M. (2010). Extended-Connectivity Fingerprints. J. Chem. Inf. Model..

[47] Guha R., Van Drie J.H. (2008). Structure−Activity Landscape Index: Identifying and Quantifying Activity Cliffs. J. Chem. Inf. Model..

[48] González-Medina M., Méndez-Lucio O., Medina-Franco J.L. (2017). Activity Landscape Plotter: A Web-Based Application for the Analysis of Structure–Activity Relationships. J. Chem. Inf. Model..

[49] Bemis G.W., Murcko M.A. (1996). The Properties of Known Drugs. 1. Molecular Frameworks. J. Med. Chem..

[50] Berthold M.R., Cebron N., Dill F., Gabriel T.R., Kötter T., Meinl T., Ohl P., Sieb C., Thiel K., Wiswedel B., Preisach C., Burkhardt H., Schmidt-Thieme L., Decker R. (2008). KNIME: The Konstanz Information Miner. Data Analysis, Machine Learning and Applications.

[51] Medina-Franco J.L., Naveja J.J., López-López E. (2019). Reaching for the Bright StARs in Chemical Space. Drug Discov. Today.

[52] Naveja J.J., Medina-Franco J.L. (2019). Finding Constellation in Chemical Space Trough Core Analysis. Front. Chem..

[53] Naveja J.J., Vogt M., Stumpfe D., Medina-Franco J.L., Bajorath J. (2019). Systematic Extraction of Analogue Series from Large Compound Collections Using a New Computational Compound–Core Relationship Method. ACS Omega.

