# Supplementary figures and images for: Tubulin Inhibitors: A Chemoinformatic Analysis Using Cell-Based Data
Source: Molecules. 2021 Apr 24;26(9):2483. doi: 10.3390/molecules26092483 (PMC8123128; doi:10.3390/molecules26092483)

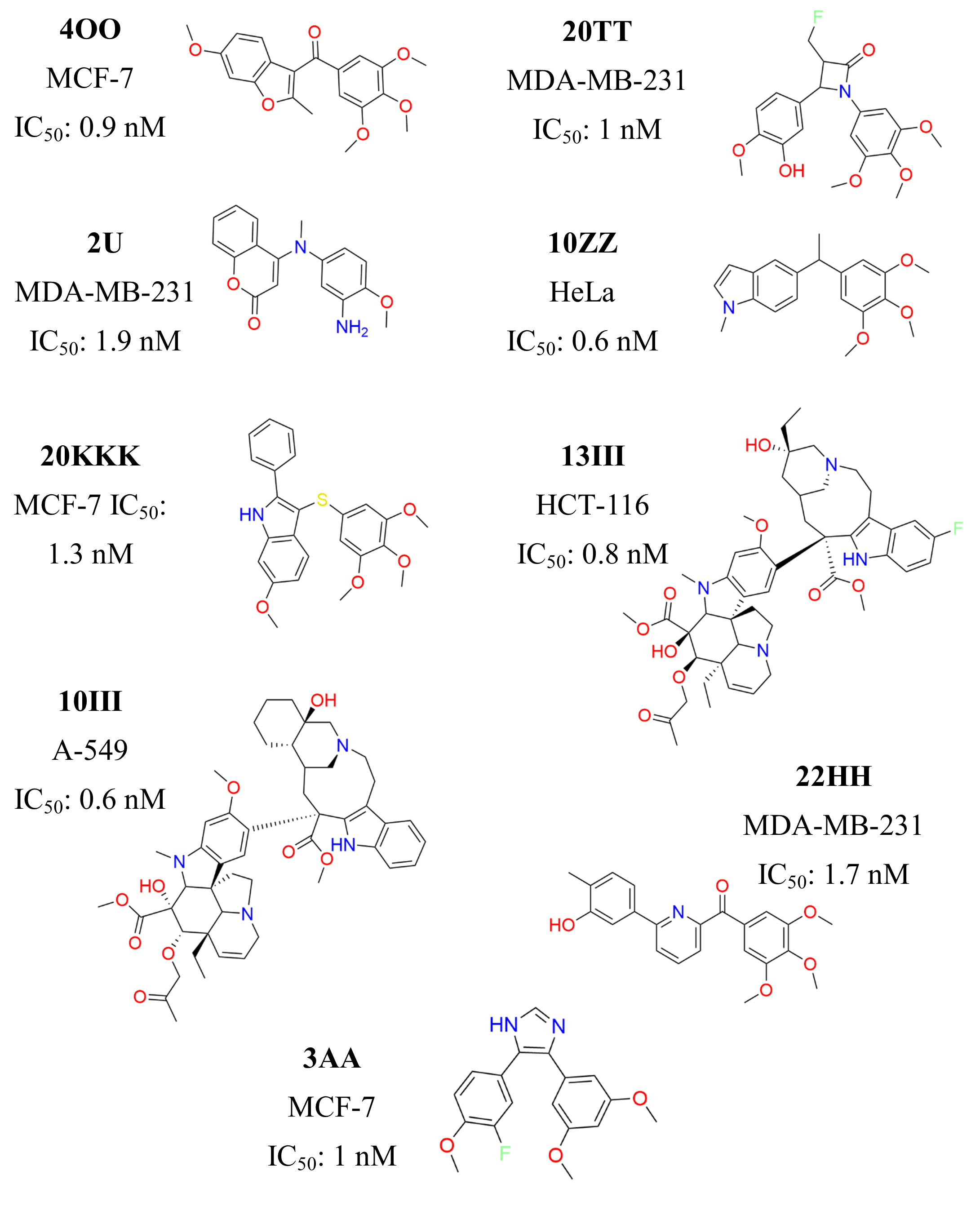

Supplement: Supplementary file 1 [file molecules-26-02483-s001.zip › Figure_S1.png]

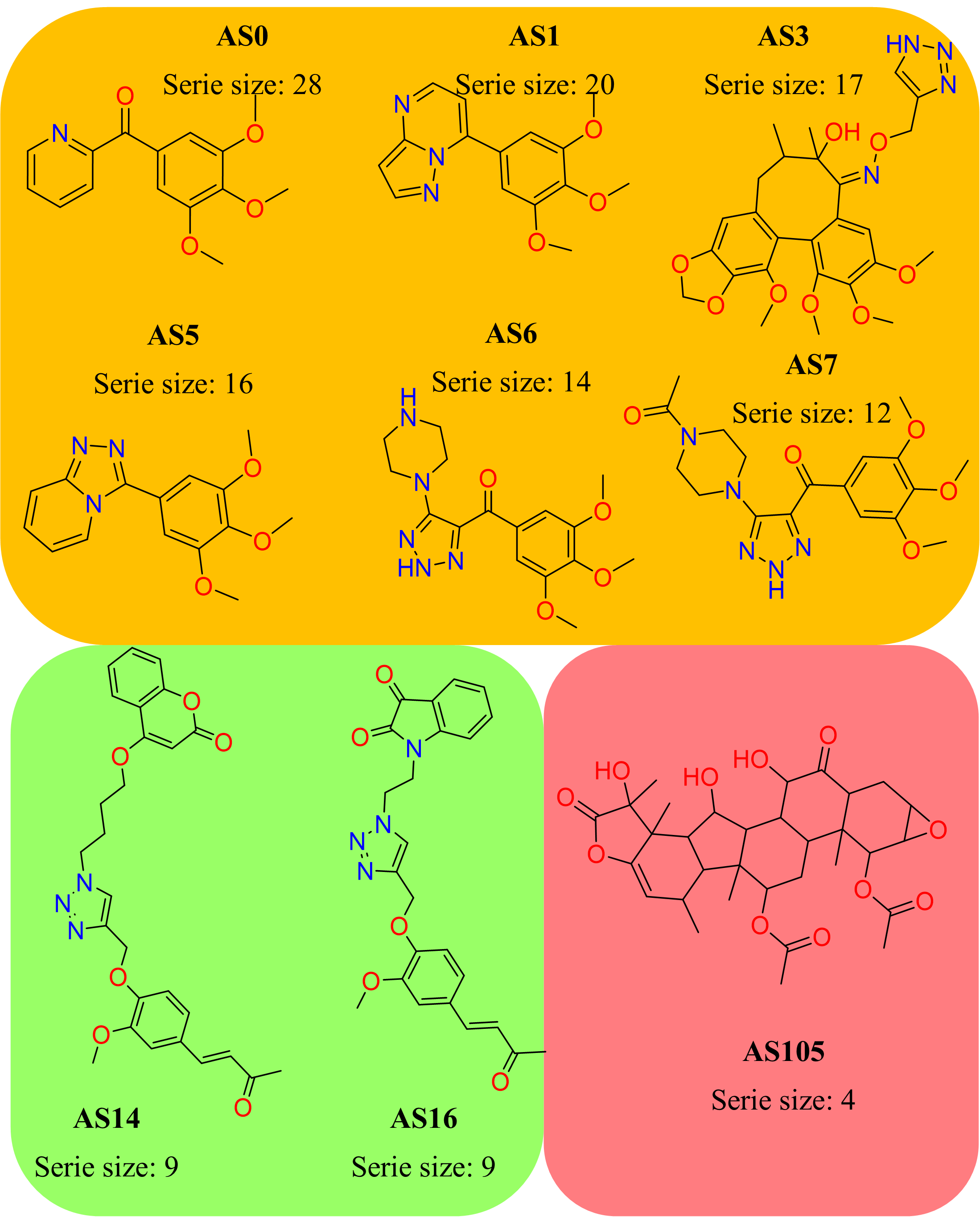

Supplement: Supplementary file 1 [file molecules-26-02483-s001.zip › Figure_S2.png]
